# Ventricle Boundary in CT: Partial Volume Effect and Local Thresholds

**DOI:** 10.1155/2010/674582

**Published:** 2010-05-17

**Authors:** Ihar Volkau, Fiftarina Puspitasari, Wieslaw L. Nowinski

**Affiliations:** Biomedical Imaging Laboratory, Agency for Science, Technology and Research (A*STAR), 30 Biopolis Street, #07-01, Matrix, Singapore 138671

## Abstract

We present a mathematical frame to carry out segmentation of cerebrospinal fluid (CSF) of ventricular region in computed tomography (CT) images in the presence of partial volume effect (PVE). First, the image histogram is fitted using the Gaussian mixture model (GMM). Analyzing the GMM, we find global threshold based on parameters of distributions for CSF, and for the combined white and grey matter (WGM). The parameters of distribution of PVE pixels on the boundary of ventricles are estimated by using a convolution operator. These parameters are used to calculate local thresholds for boundary pixels by the analysis of contribution of the neighbor pixels intensities into a PVE pixel. The method works even in the case of an almost unimodal histogram; it can be useful to analyze the parameters of PVE in the ground truth provided by the expert.

## 1. Introduction

Feature analysis is an important process in biomedical image processing. It allows extracting anatomical structures of interest (e.g., ventricles from neuroimages, liver from abdominal images, or blood vessels) or abnormalities corresponding to particular diseases (e.g., stroke, bleeding, or tumor). Various techniques have been proposed to perform feature extraction in biomedical images. One of the common techniques is to apply thresholding; a survey and comparison of these techniques is in [1].

Some methods are purely based on intensity histogram of the image, where the threshold value may be determined by several ways: by minimizing the within-class variance of the histogram in Otsu's method [2], which was further improved in [3, 4], or based on the median of the histogram with assumption that the percentage of object pixels is known [5], based on the histogram valley [6], or based on the histogram concavity analysis [7]. Other methods calculate the threshold by maximizing the entropy of the histogram [8, 9], or can be based on the condition that the thresholded image should have the same moments as the original image [10]. 

The abovementioned techniques only provide a global threshold, in which the spatial information among the neighborhood pixels is not taken into consideration. In medical images, it is pretty common to encounter intensity inhomogeneity, and in this case global thresholding alone may not be able to provide a good result. 

In the field of MR images, there are numerous algorithms to segment the ventricular region based on different techniques, such as intensity-based method [11, 12], model-based method [13, 14], or combination of them [15, 16]. However, these methods may not work well when they are applied to CT neuroimages because of high slice thickness and noise.

Several studies have proposed algorithms for CT neuroimage segmentation. Methods in [17, 18] propose the segmentation of CT images based on k-means with the expectation-maximization (EM) clustering and 2D Otsu threshold segmentation with the particle swarm optimization (PSO) algorithm, respectively, which separate the brain region into three clusters: brain matter, CSF, and abnormal regions. Another study in [19] applied a two-stage fuzzy C-means (FCM) algorithm to extract brain tissue and exclude CSF and skull. These three methods do not provide a specific segmentation of the ventricular region. A different approach is introduced in [20] whereby two-stage segmentation was performed. The ventricle is initially segmented using either Iterated Conditional Model (ICM) or Maximum A Posterior Spatial Probability (MASP), and the result is further refined using a template-matching technique. 

We propose a two-stage algorithm based on statistical consideration to find the parameters of the partial volume effect (PVE) pixels distribution. These stages include global thresholding and local analysis of the pixels to refine the boundary of the ventricles.

## 2. Method

We consider the intensity histogram of the current slice as a Gaussian mixture model (GMM) [21] with the density function

(1) $$f\left(x\right)=\overset{N}{\underset{i=1}{\sum }}\pi_{i}f_{i}\left(x\right),$$

where *f*_*i*_(*x*)  are the *i*th-component Gaussian probability density functions, *π*_*i*_ are weights,  ∑_*i*=1_^*N*^ *π*_*i*_ = 1, 0 ≤ *π*_*i*_ ≤ 1, and *x* is intensity of the pixel. Particularly, two out of all components are the white and grey matter (WGM) and the cerebrospinal fluid (CSF). Let us denote their distributions as *f*_1_ and *f*_2_, respectively:

(2) $$\begin{array}{c} f_{1}\left(x\right)=N\left(x\;|\;\mu_{1},\sigma_{1}\right),\\ f_{2}\left(x\right)=N\left(x\;|\;\mu_{2},\sigma_{2}\right), \end{array}$$

where *N* is Gaussian probability density function with mean value  *μ* and standard deviation *σ*:

(3) $$N\left(x\;|\;\mu ,\sigma \right)=\frac{1}{\sigma \sqrt{2\pi }}\exp\;\left(-\frac{{\left(x-\mu \right)}^{2}}{2\sigma^{2}}\right).$$

Knowing the standard Hounsfield units (HUs) for the WGM and the CSF, we consider the range of intensities for our analysis from 0 to 75 HUs.

We employ the GMM with 3 components. To estimate the parameters of the GMM (1), the EM algorithm [22] is used. The parameters of the CSF and the WGM distributions are used to find the distribution of the pixel on the boundary between these two tissues, which hereafter we denote as PVE distribution.

Let us suppose that the part of the pixel filled with CSF is *α* and the part filled with other tissues is (1 − *α*). To find the PVE distribution, we calculate convolution of two Gaussians for CSF and WGM, respectively:

(4) $$g\left(z\right)=g\left(\left(1-\alpha \right)x+\alpha y\right)=\overset{}{\underset{x}{\int }}f_{1}\left(z-\left(1-\alpha \right)x\right)f_{2}\left(\alpha x\right)\;dx.$$

Here *z* is a sum of random values:

(5) z=(1−α)x+αy,

where *x* and *y* are independent random variables having Gaussian distributions, and the *α* part of pixel has the random value from the CSF distribution and (1 − *α*) from WGM. Taking into account formulas (2)–(5) and the fact that the sum of two independent normal random variables again has a normal distribution, we can find the parameters of the resulting normal distribution:

(6) $$\mu_{g}=\left(1-\alpha \right)\mu_{1}+\alpha \mu_{2},$$

(7) $$\sigma_{g}=\sqrt{{\left(1-\alpha \right)}^{2}\sigma_{1}^{2}+\alpha^{2}\sigma_{2}^{2}},$$

where *μ*_*g*_  and *σ*_*g*_  are the mean and standard deviations of the PVE distribution as a result of convolution between CSF and WGM distributions. 

Let us assume that, if a part of CSF in a pixel is at least *α*, the pixel is assigned to the CSF class. The parameter *α* may depend on a person (expert) who marks the ground truth (GT). To estimate *α*, we consider ventricles marked in the GT, get their boundary pixels (i.e., pixels with the PVE), and find the mean value *μ*_*g*_ for the distribution of these pixel intensities. Then, using formula (6), value *μ*_*g*_, and the parameters of the CSF and the WGM distributions, we can estimate the value of  *α*. Hereafter we denote the value specific for the expert as  *α*_0_. Though *α*  is included into formulae (6) and (7), we use formula (6) to find *α*_0_ because it provides a lesser error: calculation of standard deviation uses the mean value of a sample, so the magnitude of error of standard deviation may be higher. 

In the case if we do not have GT, we can assume that  *α*_0_ = 1/2; that is, the pixel belongs to CSF if at least 1/2 of the pixel is CSF.

The most probable value for the pixel with PVE corresponds to *μ*_*g*_. Let us denote this value as threshold *T*_PVE_. We assign pixels to the CSF class if their intensities are not above *T*_PVE_. Pixels adjacent to the CSF ones are analyzed during the second phase of the algorithm by calculation of contribution of neighboring pixels intensities into a pixel under consideration. In other words, we calculate the possibility for the pixel to have at least an *α* part of CSF taking into account the value of the nearby pixels. 

Let us assume now that we extract a connected component corresponding to the ventricle and find the boundary pixels of this component (the component contour). Let us denote *B* as a set of pixels which are adjacent to the boundary; that is, one pixel width layers inside and outside the boundary. Let us assign the value *e*(*x*) of the image force to the pixels *q* with intensity *x*:

(8) $$\begin{array}{cc} & e\left(x\right)\\ & =\{\begin{array}{cc} 0 & \text{if}\;\text{the}\;\text{pixel}\;q\;\text{is}\;\text{outside}\;B,\\ \max\;\left(\frac{\alpha_{0}N\left(x\;|\;\mu_{1},\sigma_{1}\right)}{N\left(\mu_{1}\;|\;\mu_{1},\sigma_{1}\right)},\beta \;\right), & \text{otherwise}. \end{array} \end{array}$$

The higher the value of *e*(*x*), the more probable the pixel is on the boundary of a ventricle. The first item *α*_0_ *N*(*x* | *μ*_1_, *σ*_1_)/*N*(*μ*_1_ | *μ*_1_, *σ*_1_) is a normalized, between 0 and *α*_0_, probability for pixel to be a mixture of the CSF and WGM. The second item *β* is a possible share of CSF in the PVE pixel. Let us consider three consecutive pixels *p*, *q*, and *r* along some direction (we used 8 directions corresponding to 8-connectivity), and denote their intensities *g*(*p*), *g*(*q*) = *x*, and *g*(*r*). Let us assume that pixel *p* ∈ *B* and is marked as CSF, while *q* and *r* are not (i.e., *g*(*q*) and *g*(*r*) are greater than *T*_PVE_). If any of these assumptions is not satisfied, we set *β* = 0; otherwise we find the value *β* from the equation similar to formula (6):

(9) g(q)=(1−β)g(r)+βg(p).

In the case *β* ≥ *α*_0_, the inequality *g*(*q*) ≤ (1 − *α*_0_)*g*(*r*) + *α*_0_ *g*(*p*) is satisfied, and we can assume that the pixel *q* (which is in between CSF pixel *p* and non-CSF pixel *r*, and which has more than *α*_0_ parts of CSF) also belongs to the ventricle and is situated on its boundary.

Anatomically, the boundary of the ventricle should be a smooth curve, so the pixelwise contour should be smoothed to look realistic. We use snake [23] with the image force defined by (8) to smooth the contour. For energy functional of the snake, the weight coefficients for continuity term, curvature term, and image force were taken as 1, 1.5, and 1.2, respectively.

## 3. Results

**Figure 1:**
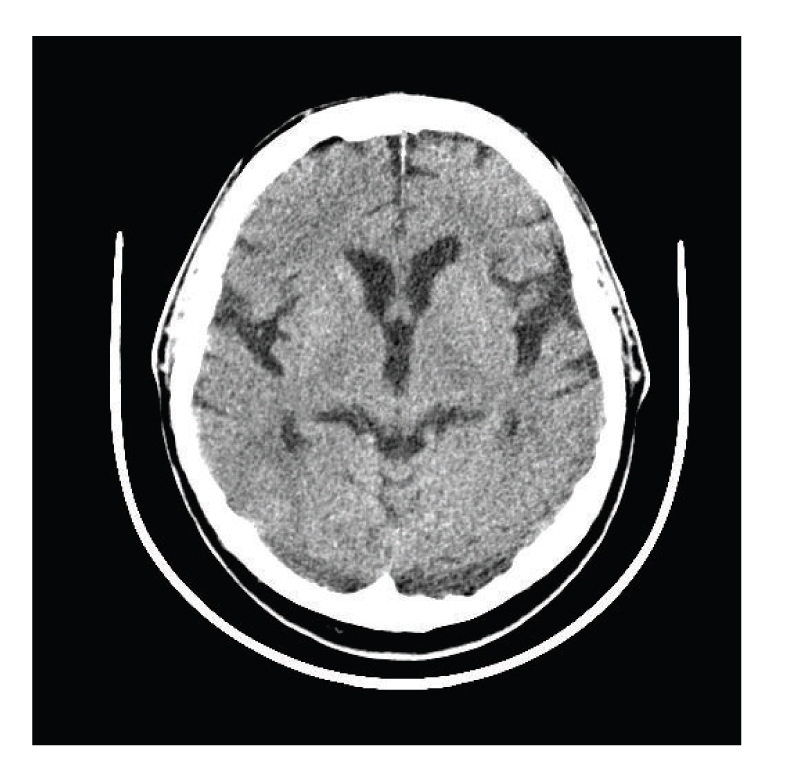
CT slice to extract ventricles.

**Figure 2:**
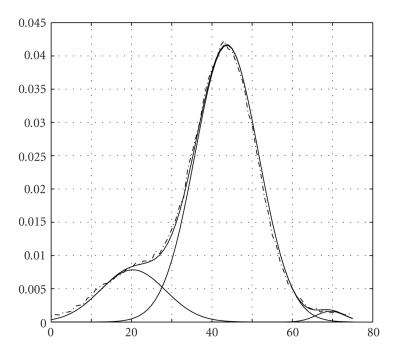
GMM with 3 components for CT slice; HUs from 1 to 75 are given, and the vertical axis is the probability.

We have applied the proposed algorithm to CT scans. To illustrate a typical behavior of the algorithm, we present a case below. The original CT slice is shown at Figure 1 and its histogram is in Figure 2. The dash-dot line corresponds to the original histogram, and the solid lines correspond to the components of the GMM and to the sum of all of the components. We use the GMM with 3 components. It can be seen that the approximation by 3 Gaussians provides a reasonable fit to the original histogram. 

**Figure 3:**
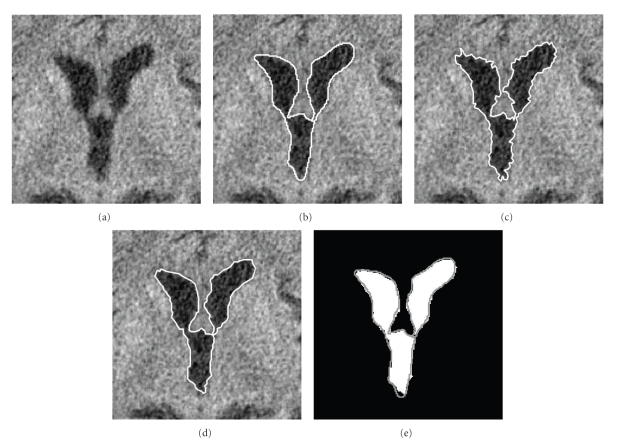
Ventricle extraction: (a) original ventricles in CT, (b) the contour of the GT marked, (c) the result of the thresholding, (d) result of smoothing using snake, and (e) the contour (grey line) of ventricle received by algorithm overlapped over the GT (white area).

The original image, the ground truth, and the results of the initial step and the second phase are shown in Figure 3.

For Figure 3(a) the changes of the intensities inside and on the boundary of the darker spots in the image (i.e., within the ventricular system) may be because of acquisition noise, choroid plexus, or partial volume effect due to the high slice thickness, or something else. Our algorithm calculates and finds the probable boundary points of the ventricles. Using the GT marked by the expert, we found that the parameter *α*_0_ = 0.6. We do not do the statistical analysis based on comparison with the rater's generated ground truth because the main target of the rater was to delineate the ventricles, but not to provide some systematic and nonsubjective way to determine the belonging of the boundary pixels to inner or outer parts of the ventricles. 

## 4. Discussion

The Gaussian Mixture Model (GMM) can be used for the initial segmentation of the CT images [17] into CSF and WGM under the assumption that the distributions of intensities for these peaks are Gaussians, and pixel values are independent random variables. Knowing the standard range of Hounsfield units (HUs) for the WGM and CSF, we can find the parameters of these Gaussians while using the Bayesian approach to find the threshold to separate these two peaks (it corresponds to the point of the cross-section of these Gaussians). Partial volume voxels contain multiple tissue types; in such a case in the GMM each tissue is not represented by a single Gaussian. It makes it difficult to use the traditional Bayesian classification if two components are close to each other or have an overlap and the distinction between them is not obvious. In CT, the ranges of intensities from CSF and WGM are usually overlapped, which can even result in a unimodal distribution of the sum of these distributions. Characteristics of the mixture of the CSF and WGM are also changing along the ventricular boundaries. 

In the case of the ventricle boundary, anatomically we have two types of media, CSF, and WGM, which do not mix with each other, but because of the PVE, we may have a mixture of intensities. The same is true when CSF is a neighbor for a calcified structure. It leads to the appearance of additional distribution(s) corresponding to these mixtures. As the CSF and the WGM both are assumed to be Gaussians, their mixture should also have a Gaussian distribution.

For the GMM we use the EM algorithm [22] to estimate the vector of unknown parameters of components in the GMM. The likelihood function for the mixture model usually has multiple local maxima [21], and there is a question about which root of the likelihood equation corresponds to the global maximum of the likelihood function. According to [24], the ventricle to brain volume ratio for normal population is 1.29 ± 0.4%. The pixels on the boundary of ventricles have even smaller ratio to the volume of the brain; hence, the peak, corresponding to the PVE, is almost undistinguishable in the brain tissue intensities histogram and may be completely disguised by noise. Additional source of possible errors in identification of parameters of the PVE distribution may be the incorrectly chosen number of classes in the GMM, which leads to the systematic bias in the mean and standard deviations of extracted parameters (the same effect occurs when using the truncated distributions).

The parameters of the PVE distribution may be estimated indirectly by the EM algorithm for the GMM. First, we extract more pronounced peaks, corresponding to WGM and CSF, which we assume to be Gaussian distributions with the weight coefficients *π*_1_ and *π*_2_, respectively. Then, their mixture (4) should also be a Gaussian distribution with parameters (6) and (7) as a convolution of two normal distributions. We can expect that the weight coefficient for the PVE distribution will be lesser than  *π*_1_∗*π*_2_, because only the boundary of CSF and WGM pixels participates in this mixture, and this boundary is negligible in comparison with the area of the tissues and ventricles by themselves; hence, the parameters of the PVE distribution can hardly be extracted directly from the histogram in the correct way. 

As for the boundary of the ventricle, several observations can be done. The thickness of the ventricle boundary cannot be more than one pixel. The boundary pixel in such a case can either belong to one of two tissue classes or be the mixture of these 2 classes. Because of high slice thickness, the PVE effect may not be pronounced only on the boundary, but inside the ventricle as well; that is why we assign pixel to the CSF class if its intensity is below *μ*_*g*_.

To mark the GT, the expert uses image data and anatomical knowledge. Anatomically the contour of the ventricle should be a smooth curve; that is why the contour marked might not follow the edge of the thresholded ventricle. To take into account the anatomical property, we use snake to smooth the boundary of the ventricle using an image force as the function *e*(*x*) defined by (8). A higher value of *e*(*x*) reflects a higher probability of pixel to lie on the ventricle boundary.

A two-stage algorithm gives a chance to discriminate global and local properties using the analysis of vicinity of the current contour point under consideration. Belonging of the pixel to the specific group in the overlapped area of histogram cannot be identified not taking into account some additional spatial information. Therefore, probably, the dynamic programming-type techniques may be applied, for recalculation of the moving boundary of the ventricle or the tissues. In such a case, we have information about probabilistic distributions in two media and should formulate the objective function regarding how to estimate the risk of adding the pixel to the first or the second class, taking into account the connectivity and clusterization existing in the VOI. 

The results of boundary analysis will vary with the change of the value of *α*. Let us analyze some well-known algorithms from the point of view of mixture of two distributions. The algorithm [25] fits a rectangle to the profile, setting the width so that the area under the rectangle is equal to the area under the profile. If a vessel's intensity is fitted by the Gaussian shape and the background has the uniform intensity, then the cutoff thresholds are $\mu \pm \sigma \sqrt{\pi /2}$; that is, the pixel is considered as belonging to a vessel if it has less than 10.5% of background. For the full width at half-maximum approach, the condition is to have not more than 50% of background.

A usual way to validate the results of the algorithm is to compare them with the expert's provided marks, otherwise called ground truth (GT). Different experts may mark ground truth differently. Even for the same expert the results of marking can be different depending on the time of the day or day of the week and image windowing. That is why one of the standard characteristics which can be provided for ground truth is an intraexpert and interexpert variability. 

The HUs for the CSF in CT should be approximately in the range from 0 to 21 (depending on the energy and scanner calibration). The rater's generated ground truth may not reflect correctly the pixels on the boundary of ventricle because of subjectivity of judgment and the way of marking. For example, the expert's delineations of the ventricles included intensities from −15 to almost 800 HUs. The marking of the pixel might depend on the PVE and on image contrast in the vicinity of the pixel (e.g., a calcified object adjoining the CSF can cause a few pixels to be marked as CSF even having the intensity of 800 HUs due to PVE); misclassification can be caused by the tiredness of doing the tedious work of marking hundreds and thousands of pixels. Additionally, we have representation of the pixel value in the image as an integer grayscale; the value of the pixel is received from the mathematical reconstruction algorithm and additionally the value was rounded to the integer with some possible loss of accuracy. So the value of the pixel is by itself an approximation of the value of the manifestation of the response from the tissue at the current position. The expert's marking may be based on hardly formalizable ideas and experience which also barely can have some verbalization. The ventricle boundary pixels with the same intensity may be at the same time partially included into ventricle and partially excluded from it.

There is a lack of objective criteria to estimate the precision of the GT. We can hardly describe what the optimal solution for the problem of identification of the boundary is. Comparison with the GT marked by the expert shows that the expert opinion to include or not to include the points into the cluster sometimes is nonformalizable. We cannot expect that the expert with the same accuracy and without tiredness will mark thousands of pixels individually. So the basis for the estimation of the algorithm performance and its results may not be the optimal solution, but the acceptable one, satisfying some reasonable (or at least clearly pronounced) assumptions.

Taking into account the value of *α* which corresponds to the experts' judgments may be considered as an adjustment of the algorithm of segmentation to the expert's implicit requirements.

## 5. Conclusion

The existence of the PVE makes it necessary to use not the independent random variable model but a conditional probabilistic model. We present a two-phase algorithm which is based on theoretical calculation of parameters of the PVE and takes into account the vicinity around the pixel for which the decision should be made regarding which class to join. The approach described can be used to estimate the parameter for the PVE which the expert implicitly uses to mark the ground truth.
